# Point-of-Care Ultrasound Training and Credentialing for mid-late Career Emergency Physicians: Is it worth it?

**DOI:** 10.24908/pocus.v6i2.14891

**Published:** 2021-11-23

**Authors:** Courtney M Smalley, Erin L Simon, Mckinsey R Muir, Fernando Delgado, Baruch S Fertel

**Affiliations:** 1 Cleveland Clinic Health System; 2 Akron General Medical Center

**Keywords:** Credentialing, Point-of-Care Ultrasound, Late-Career Physician, Competency, Privileging, Emergency Medicine

Dear Editor:

Point-of-care ultrasound (POCUS) is becoming more prevalent in community emergency medicine (EM) practice with the current American College of Emergency Physician guidelines recommending POCUS training for all graduates from United States based residency programs as well as support for POCUS privileging by the American Medical Association 1, 2, 3. However, in a recent survey of nonacademic EDs, it was found that most providers lack US training, credentialing, and quality assurance (QA) assessments of their POCUS studies 4. In 2017, our healthcare system embarked on a system-wide credentialing process for POCUS to credential community physicians with little to no POCUS training 5. After successful implementation and completion of the program in 2018, we sought to examine the group of mid to late career emergency physicians to assess how these physicians utilized POCUS longitudinally in their practice after credentialing and to assess QA issues with image acquisition and image interpretation in this cohort. 

We performed a retrospective qualitative review of POCUS studies performed after a POCUS credentialing initiative for EM physicians was completed across 11 non-academic hospitals from January 1, 2017 to July 1, 2018, 5. Credentialing in “Basic” POCUS required a completion of a dedicated POCUS course as well as a practice-based competency pathway which included Focused Assessment of Sonography in Trauma (FAST), aorta, and central line ultrasound training (Table 1). A practice-based pathway is defined as a pathway for practicing EM physicians who completed residency without POCUS training who undergo a series of introductory training, small group hands-on instruction, and practice with image acquisition and image interpretation of POCUS exams 1. To complete the program within our healthcare system, EM physicians must complete the minimum number of exams (60 scans total in 3 modalities) to gain privileges in “Basic” POCUS. While they could complete other scan types during their credentialing period, they were not eligible to become credentialed in other scan types until the “Basic” POCUS practice-based pathway was complete. For the physicians who successfully completed the practice-based pathway for competency by June 30, 2018, we reviewed the number of studies performed, the types of POCUS studies performed, and quality issues (image acquisition, image interpretation, and labeling of images) for 28 months after POCUS training. Each physician was designated by years from residency graduation as early-career (1-10 years), mid-career (11-20 years), or late (21 years or greater).

**Table 1:** Basic Point-of-Care Ultrasound Privileges

**Credentialing Tier**	**Applications**	**Number of Scans Required**
Basic Ultrasound (all scan types required for completion)	General applications: focused assessment with sonography in trauma (FAST), US guided venous access placement, abdominal aorta aneurysm (AAA)	FAST: 25 scans AAA: 25 scans Central Line: 10 scans
Requirements for Point-of-Care Ultrasound Study 1. Adequate image acquisition 2. Adequate image interpretation 3. Appropriate labeling of each image		

At the start of the program in January 2017, 26% (28/108) of community EM physicians were without POCUS privileges. 46% (13/28) physicians completed the program and became credentialed in “Basic” POCUS exams. The mean number of years of this group since residency graduation was 19 years, IQR 19.25 (13,25.5). 1 physician was excluded from the cohort as he left the healthcare system prior to July 1, 2018, when formal privileges took effect. From July 1, 2018 – Nov 1, 2020, a total of 379 scans were performed by the 12 physicians. The mean number of scans performed per physician was 31. Three physicians performed zero scans. A total of 45% (172/379) of exams performed were eligible for documentation and billing for Basic POCUS privileges: FAST (N=158), aorta (N=13), central line (N=1), (Figure 1). Quality issues of inadequate image acquisition were identified on 17.7% (28/158) of FAST exams and 15.4% (2/13) of Aorta exams. There were no incidences of inappropriate image interpretation. 78% of studies were labeled appropriately.

**Figure 1:**
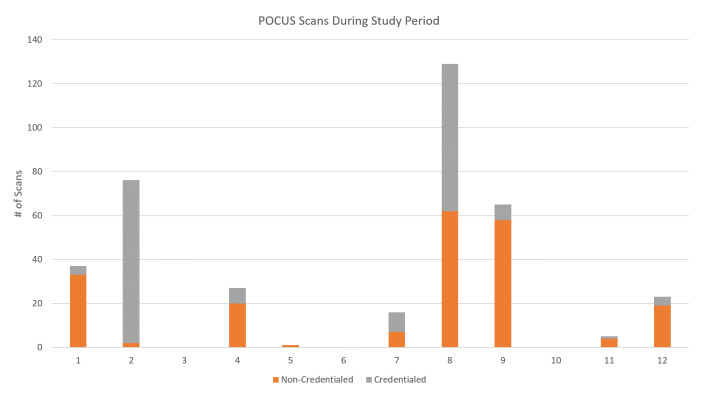
Credentialed and Non-Credentialed Physician Scans Performed after Completion of Basic POCUS Credentialing Program. Study Period: July 1, 2018 – November 1, 2020. Credentialed Scans defined as POCUS studies for which the physician has privileges and for which the study was a billable study. Non-Credentialed Scans defined as scans in other POCUS modalities for which the physician did not have privileges and the exam was a non-billable study.

A practice-based pathway to achieve POCUS credentialing for physicians without any training or experience in POCUS yielded very few credentialed billable POCUS studies. The physicians in this cohort had a median of 19 years since residency graduation with only physician in the cohort considered early-career. Overall, there were no incidences of inappropriate image interpretation but not all exams had appropriate image acquisition and labeling. 

Our results raise the question of whether training of mid to late career emergency physicians in these “basic” POCUS studies is useful. We embarked on this retrospective review to examine this question as the amount of resources and time needed to train this cohort of physicians was significant. All of these physicians had no experience in POCUS. While we were able to put them through a standardized course to teach the introductory concepts of POCUS and become privileged in 3 POCUS exams, we found that very few physicians actually utilized POCUS longitudinally after achieving competency and privileges in these studies. Financial and resource implications include the time of the dedicated POCUS trainer, review of all studies while the physicians were completing a practice-based pathway for credentialing, and oversight as they practiced clinically in their community sites with a quality assurance program. 

Those points being considered, if only one patient had a shorter length of stay or was transferred to definitive care for trauma or aortic catastrophe based on POCUS training, then the training of mid to late career EM physicians in Basic POCUS may be worth the time and resource investment. Further investigations into the use of POCUS amongst late adopters of mid to late career EM physicians should be examined to determine how programs such as this affect resource utilization within a department, outcomes of patient care, and financial implications.

## Disclosures

None.
